# One-step electrospinning route of SrTiO_3_-modified Rutile TiO_2_nanofibers and its photocatalytic properties

**DOI:** 10.1186/s11671-017-2130-9

**Published:** 2017-05-25

**Authors:** Weijie Zhao, Jing Zhang, Jiaqi Pan, Jianfeng Qiu, Jiantao Niu, Chaorong Li

**Affiliations:** 1 Department of Physics and Key Laboratory of ATMMT Ministry of Education, Zhejiang Sci-Tech University, Hangzhou, 310018 People’s Republic of China; 2 School of Medical and Pharmaceutical Engineering, Taizhou Vocational & Technical College, Taizhou, 318000 People’s Republic of China

**Keywords:** Electrospinning, Heterojuction, Photocatalyst, Recycling

## Abstract

The SrTiO_3_ modified rutile TiO_2_ composite nanofibers were synthesized by a simple electrospinning technique. The result of XRD, SEM and TEM indicate that the SrTiO_3_/TiO_2_ heterojuction has been prepared successfully. Compared with the TiO_2_ and SrTiO_3_, the photocatalytic activity of the SrTiO_3_/TiO_2_ (rutile) for the degradation of methyl orange exhibits an obvious enhancement under UV illumination. which is almost 2 times than that of bare TiO_2_ (rutile) nanofiber. Further, the high crystallinity and photon-generated carrier separation of the SrTiO_3_/TiO_2_ heterojuction are considered as the main reason for this enhancement.

## Background

As a prototypical semiconductor with environment friendly and high photoelectric property, Titanium oxide (TiO_2_) is widely used in optics, solar cells, sensors etc. [1–4], and also considered as a most promising photocatalyst in wastewater treatments [5], due to its low cost, highly physical-chemical stability and nontoxicity. As previous literature reported, though the anatase TiO_2_ exhibit better photocatalysis than the Rutile TiO_2_, but the band gap of anatase TiO_2_ (3.2 eV) is wider than the rutile TiO_2_ (3.0 eV), which may restrict the luminous energy utilization ratio in photocatalytic application. What’s more, compare with the metastable anatase TiO_2_, the rutile TiO_2_ exhibit more highly physical-chemical stability, which is beneficial for cyclic utilization in pollution treatment. With these unique advantages, how to improve the photocatalytic efficiency of the rutile TiO_2_ would be a significant issue. As known, the photocatalysis mainly depend on specific surface area or mobility and lifetime of photon-generated carriers, so lots of work have been reported. For specific surface area, lots of excellent morphology have been prepared, such as nanosheets [6], nanobelts [7], nanorods [8], nanofibers [9], and microflowers [10], all of them shows a inspiring results [11–14]. On the other hand, the surface noble metal modified or preparation of heterostructure are considered as useful ways to adjust the band structure for improving the mobility and lifetime of photon-generated carriers. However, compared with the high cost of the noble metal modified, the heterostructure is deemed as a efficient-low cost way. Lots of relevant researches have been reported, such as ZnO/TiO_2_ [15–17], CdS/ZnO [18–20], CeO_2_/graphene etc [21]. Among those semiconductors, the strontium titanate (SrTiO_3_) has catched researchers attention due to the thermal stability and resistance to photocorrosion [22], and has been extensively applied in H_2_ generation [23], removal of NO [24], water splitting [25], and photocatalyst decomposition of dye [26–28]. In particular, as heterostructures composite photocatalyst attracted more attention, such as, Core-shell SrTiO_3_/TiO_2_ and heterostructures SrTiO_3_/TiO_2_ had showed much higher photocatalytic activity than the pure TiO_2_, which is attributed to heterostructures promote the separation of photogenerated carriers [29, 30]_._ So the SrTiO_3_ is considered as a good candidate for coupling with the anatase phase of TiO_2_ for adjusting the band structure to enhance its photocatalytic activity_._ However, there are rare reports about the SrTiO_3_-modified rutile TiO_2_ composites nanofibers for the degradation of dye pollutants because of the cumbersome process, so how to simplify the preparation of SrTiO_3_/TiO_2_ nano-heterojunction would be an important issue for its practical application. As known, the electrospining is a convenient and efficient method to prepared nanomaterials, which could easily prepare the precursor into nanofibers at the prelusion and then form to series of nanostructure in subsequent annealing, which has been reported in lots literatures [31–36].

In the present study, we report on a simple one-step synthesis of SrTiO_3_ modified rutile TiO_2_ nano-heterojunction with high photocatalysis via the electrospinning. Then the mechanism of the photocatalytic enhancement of the heterojuction has been studied.

## Methods

### Materials

Analytical grade acetic acid, N,N-Dimethylformamide (DMF, Aladdin, 99.5%), Tetra butyl titanate (TBT, Aladdin, 99.0%), Strontium acetate (Aladdin, 99.97%), Polyvinylpyrrolidone (PVP, M_W_ = 1,300,000) were obtained from Shanghai Macklin Biochemical Co. Ltd.

### Preparation of SrTiO_3_/TiO_2_ (rutile) Composite Nanofiber

**Fig. 1:**
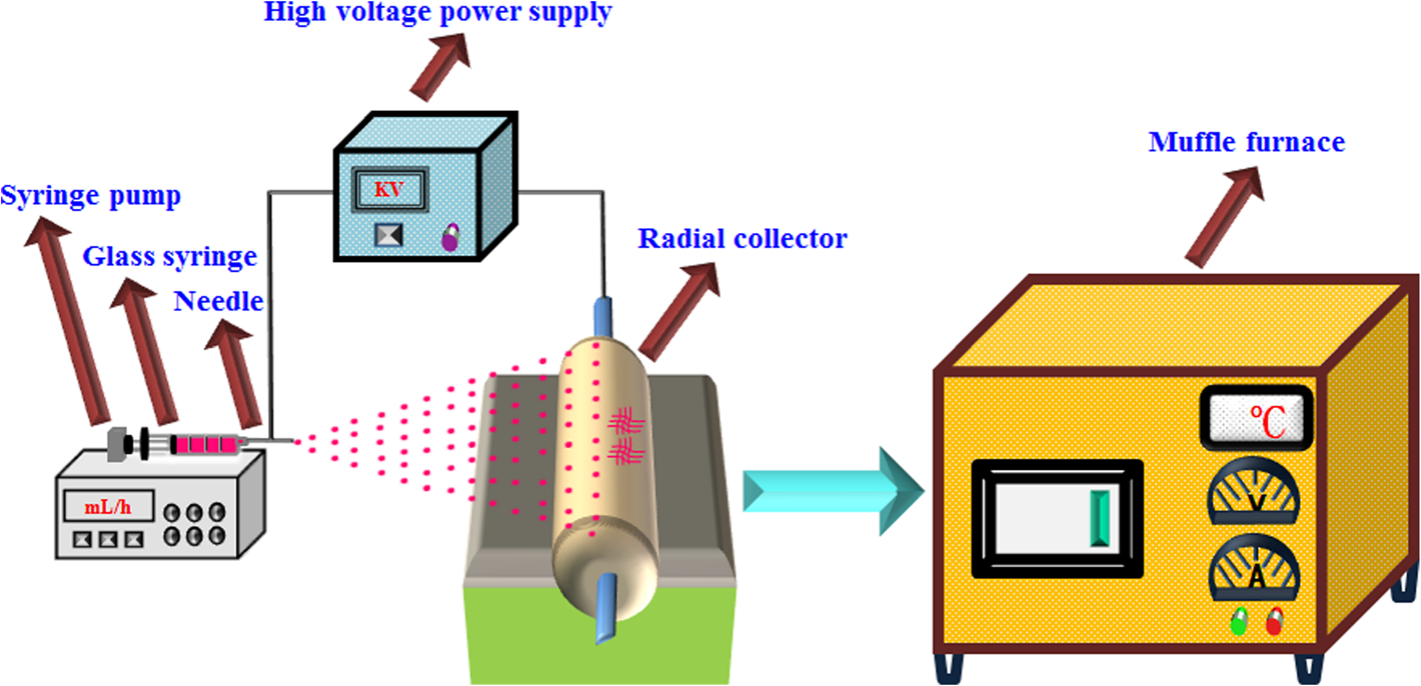
Schematic diagram of the preparation process of photocatalyst

SrTiO_3_/TiO_2_ (rutile) composite nanofibers was synthesized by directly electrospinning with subsequent calcinations method are shown in Fig. 1. Firstly, the precursor solution was prepared by dissolving 2.2 g PVP into 8 mL DMF and 2 mL acetic acid. After stirring 8 h, 2 g of TBT was added to the precursor solution for 4 h with a magnetic stirrer. Further, a certain amount of strontium acetate was slowly added into above mixture and stirred until the solution is transparent. The prepared sol-gels were loaded in glass syringe, fitted with a 0.5 mm diameter stainless steel needle and clamped in syringe pump (0.6 ml/h, KDS-200, KD Scientific, United States). This needle is connected to the positive electrode of 15 kV (Model: ES40P-10 W, Gamma HighVoltage, United States). A distance of 15 cm was maintained between the needle tip and the grounded aluminum foil collector. During electrospinning process, the humidity was maintained at < 40%, and the ambient temperature was 20 °C. Non-woven nanofiber webs were consequently obtained at the collector and left in an oven at 80 °C drying 6 h. The electrospun nanofibers were calcined in the air at 700 °C (5 °C / min heating) for 1 h to obtain the different ration of SrTiO_3_/TiO_2_(rutile) nano-heterojuction. What’s more, a bare TiO_2_(rutile) nanofibers and SrTiO_3_ nanofibers were prepared for contrast. The different ration of SrTiO_3_ in SrTiO_3_/TiO_2_ (rutile) nano-heterojuction was 1 wt%, 3 wt%, 5 wt% and 10 wt%, and marked as ST-1, ST-3, ST-5, ST-10, respectively.

### Characterization

The surface morphology of the as-prepared samples was investigated by the Field-emission scanning electron microscope (FESEM, Hitachi S-4800) equipped with Energy- dispersive X-ray spectroscopy (EDS), and the microstructure of the as-prepared samples was observed by a transmission electron microscope (TEM, JEM-2100, 200 kV); Crystal structures of the as-prepared samples were characterized by Bruker/D8-advance with Cu Kα radiation (λ = 1.518 Å) at the scanning rate of 0.2 sec/step in the range of 10-80°. The absorption spectrum of the as-prepared samples were recorded using by a UV–visibles pectrophotometer (U-3900Hitachi).

### Measurement of photocatalytic activity

A 50 mL methyl orange (MO) solution with an initial concentration of 15 mg/L in the presence of sample(30 mg) was filled in a quartz reactor. The light source was provided by a UV − C mercury lamp (Philips Holland, 25 W). Prior to irradiation, the solution was continuously kept in dark for 30 min to reach an adsorption–desorption equilibrium between organic substrates and the photocatalysts. At given intervals (t = 10 min) of irradiation, the samples of the reaction solution were taken out and analyzed. The concentrations of the remnant dye were measured with a spectrophotometer at λ = 464 nm.

## Results and discussion

**Fig. 2:**
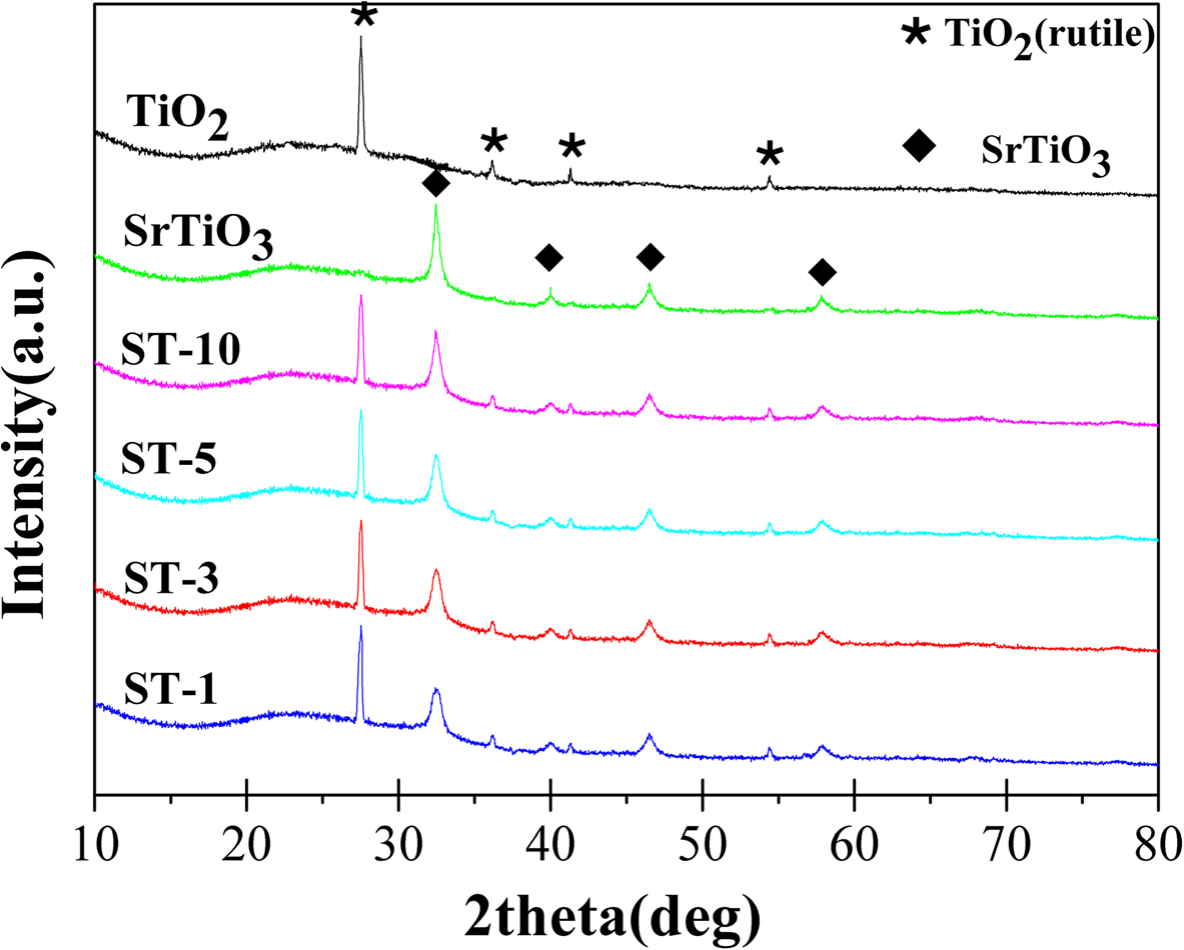
XRD patterns of the bare TiO_2_ (Rutile), bare SrTiO_3_, ST-10, ST-5, ST-3 and ST-1

Figure 2 displayed the XRD patterns of rutile TiO_2_, SrTiO_3_ and the different concentration of SrTiO_3_/TiO_2_ (rutile) nano-heterojuction. It is obvious that the diffraction peaks at 2Ɵ = 27.5, 36.1, 41.3 and 54.4 °can be indexed to the (110), (101), (111), (211) crystal planes of rutile TiO_2_ (JCPDS78-1510). The peaks at 32.4, 40.0, 46.5, and 57.8 °are attributed to the (110), (111), (200), and (211) crystal planes of Cubic SrTiO_3_(JCPDS 84–0443). The result indicates that the SrTiO_3_/TiO_2_ (rutile) composite nanofibers with higher crystallinity are successfully prepared under 700 °C sintering (Fig. 2), which may be beneficial to promote the photon-generated carrier transporting to increasing the photocatalysis.

**Fig. 3:**
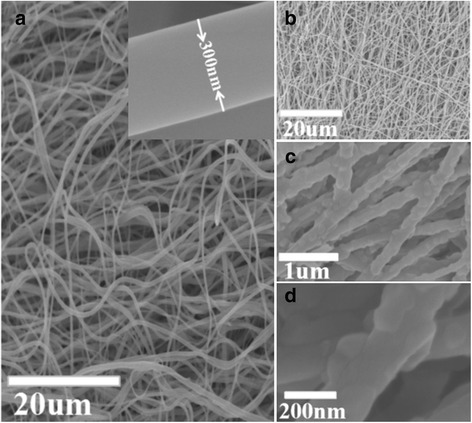
FESEM image of ST-3. **a** as-prepared ST-3, *inset*: High magnification SEM (unsintered), (**b**)-(**d**) ST-3 (sintered)

The surface morphology of the as-spun ST-3 measured by FESEM was shown in Fig. 3(a)-(d). The unsintered ST-3 preliminary composite nanofiber was illustrated in Fig. 3 (a). As shown, the surface of obtained nanofibers with diameter approximately 300 nm is smooth and continuous. Since TBT could be rapidly hydrolyzed by moisture in the air, continuous networks of TiO_2_ sols were formed in the nanofibers once they had been ejected from the needle tip [37]. As presented in Fig. 3(b), after sintering at 700 °C, the diameter of nanofibers decreased to about 200 nm and the fibers are still continuous. It’s interesting that the fiber after sintering, the nanofibers became slender and rough, which could generate much more specific surface area to increase the photocatalysis.

**Fig. 4:**
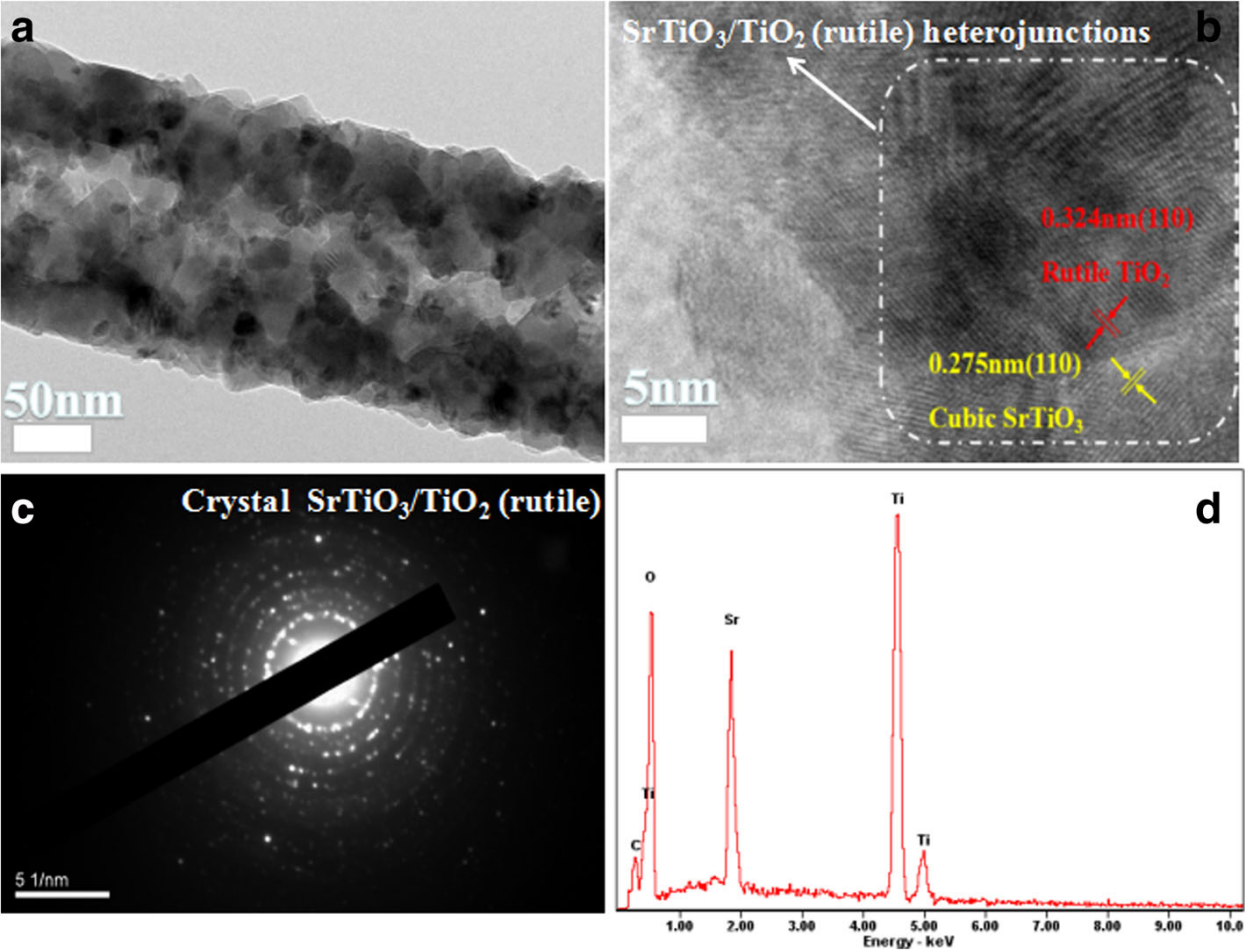
TEM image and EDS spectrum of ST-3. **a** TEM image of ST-3, (**b**) HRTEM of the delineated area of the rutile TiO_2_ and SrTiO_3,_ (**c**) SAED of ST-3, (**d**) EDS of ST-3

The TEM images provided further insight about the crystalstructure of ST-3 composite nanofibers. Figure 4a shows a typical TEM image for ST-3, which is corresponded to the SEM. HRTEM was employed to further illuminate the crystal structures of rutile ST-3 composite nanofibers. As shown in Fig. 4b, the high magnification HRTEM image reveals clearly indicates two distinctive lattice of 0.324 nm and 0.275 nm respectively, which correspond to the (110) plane of rutile TiO_2_ and the (110) plane of SrTiO_3_. This result also indicates that the nano-heterojunction have formed in the SrTiO_3_/TiO_2_(rutile) composite nanofibers (Fig. 4b), which would be beneficial to separate photogenerated electrons-holes pairs.

The selected area electron diffraction (SAED) as shown in Fig. 4c, which indicates that the nano-heterojuction owns a high crystallinity. The FESEM EDX in Fig. 4d futher confirms that ST-3 heteroarchitectures contain the Ti, Sr, O elements and corresponds to the XRD.

**Fig. 5:**
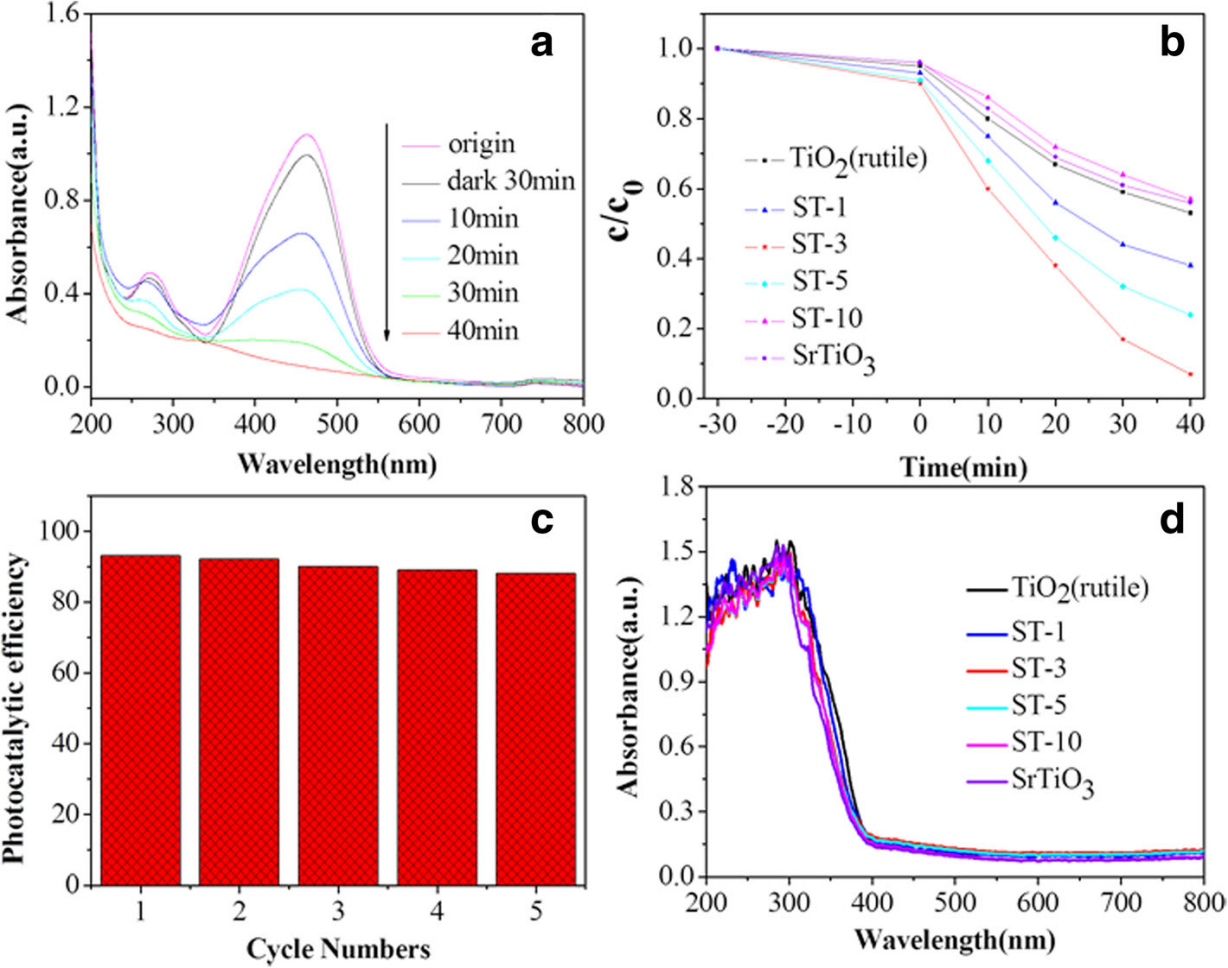
Photocatalytic activity surveying of different samples. **a** Absorption spectra of ST-3 in photocatalysis, (**b**) *Curves* of photocatalytic degradation with different products, (**c**) Recycling of the ST-3, (**d**) The UV–Vis spectra of different products

MO was used as a model dye pollutant to survey the photocatalytic activity of bare TiO_2_ (rutile), bare SrTiO_3_ and different SrTiO_3_/TiO_2_ (rutile) nanocomposites, and the results were shown in Fig. 5. After 40 min of irradiation, the rutile ST-1, ST-3, ST-5, ST-10, bare TiO_2_ (rutile) and bare SrTiO_3_ nanofibers had degraded ca. 62%, 93%, 79%, 43%, 47% and 44% of the initial MO dye, respectively (Fig. 5b). It’s interesting that, with the increasing concentration of the SrTiO_3_, the photocatalytic activity of SrTiO_3_/TiO_2_ (rutile) composite nanofibers exhibit an obviously enhancement, which indicates that the presence of the heterostructure in the composite photocatalyst is beneficial to the photocatalysis. What’s more, as shows in the Fig. 5b, when there is excess SrTiO_3_, the composites may exhibit a decreasing photocatalytic activity, which could be ascribed to that the photocatalysis of the SrTiO_3_ is much weaker than the TiO_2_, so suitable SrTiO_3_ could form the heterojuction to improve the photocatalysis efficiently but the excess SrTiO_3_ may lead an obvious decreasing.

In order to be convenient for long-term photocatalytic use in the treatment of dye wastewater, the cycling stability is one of the most important factor, and was shown in Fig. 5c. As shown in Fig. 5c, after 5 cycles, there is negligible loss of MO photodegradation, which could be ascribed to the lost of photocatalyst in centrifugal process and further illustrate that the ST-3 composite photocatalysts possess highly stability and cyclicity.

**Fig. 6:**
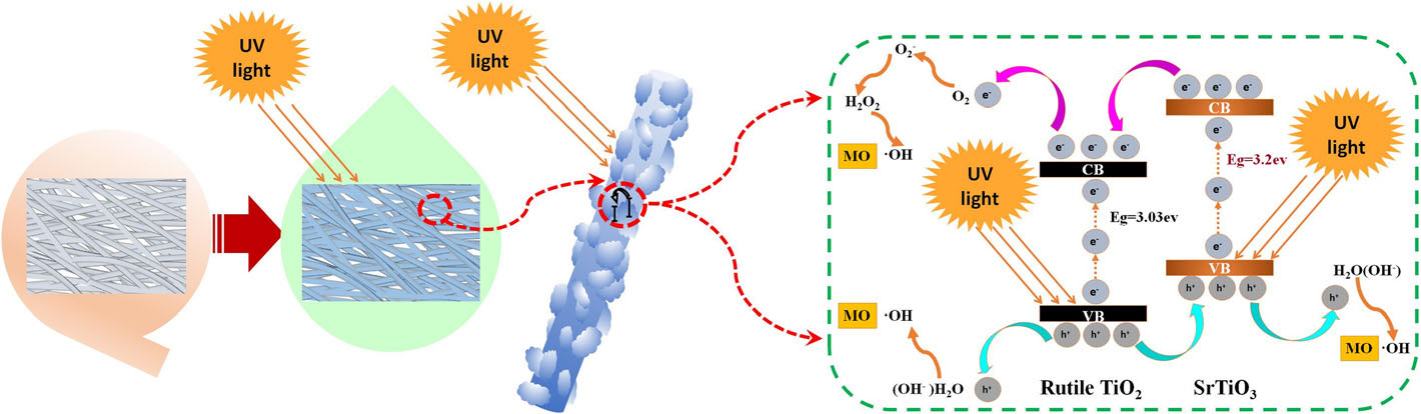
A proposed mechanism for the photocatalytic degradation of MO by the SrTiO_3_/ TiO_2_ (rutile)

As the excellent photocatalysis, the possible mechanism for the enhanced photocatalytic activity of the SrTiO_3_/TiO_2_ (rutile) composite nanofibers is very important for its further modified. As shown in Fig. 5d, the absorption of the different samples changes little, it means that the photocatalytic activity is independent with the absorption, which could be attributed to the unique nano-heterojuction. The possible mechanism is represented as follows: When UV light irradiate on surface of the composite nanofibers, both the SrTiO_3_ and the rutile TiO_2_ could generate holes (h^+^) and electrons (e^−^) as shown in (1). Then the generated electrons are immigrated from the valence band (VB) of SrTiO_3_ to conduction bands (CB) of SrTiO_3_, and further transplanted into the conduction band (CB) of rutile TiO_2._ On the other hand, the holes are transferred to VB of SrTiO_3_ from rutile TiO_2_, which could promote the charge separation efficiently to increase the lifetime of the charge carriers and enhance the efficiency of the interfacial charge transferred to enhance the photocatalytic activity of the SrTiO_3_/TiO_2_(rutile) heterostructure (Fig. 6).

Meanwhile, a probable formula of photocatalytic oxidation of methyl orange was provided as follow:

1 $$ \mathrm{SrTi}{\mathrm{O}}_3/\mathrm{T}\mathrm{i}{\mathrm{O}}_2\left(\mathrm{rutile}\right) + \mathrm{h}\upnu \to\ \mathrm{SrTi}{\mathrm{O}}_3/\mathrm{T}\mathrm{i}{\mathrm{O}}_2\left(\mathrm{rutile}\right) + {\mathrm{h}}^{+} + {\mathrm{e}}^{\hbox{-} } $$

2 $$ {\mathrm{h}}^{+} + \mathrm{O}{\mathrm{H}}^{\hbox{-}}\to \cdot p \mathrm{O}\mathrm{H} $$

3 $$ {\mathrm{h}}^{+} + {\mathrm{H}}_2\mathrm{O}\ \to \cdot p \mathrm{O}\mathrm{H} + {\mathrm{H}}^{+} $$

4 $$ {\mathrm{O}}_2 + {\mathrm{e}}^{\hbox{-}}\to \cdot p {{\mathrm{O}}_2}^{\hbox{-} } $$

5 $$ \cdotp {{\mathrm{O}}_2}^{\hbox{-} } + {\mathrm{H}}_2\mathrm{O}\to \cdotp \mathrm{O}\mathrm{O}\mathrm{H} + \mathrm{O}{\mathrm{H}}^{\hbox{-} } $$

6 $$ \cdotp \mathrm{O}\mathrm{O}\mathrm{H} + {\mathrm{H}}_2\mathrm{O}+{\mathrm{e}}^{\hbox{-}}\to {\mathrm{H}}_2{\mathrm{O}}_2 + \mathrm{O}{\mathrm{H}}^{\hbox{-} } $$

7 $$ {\mathrm{H}}_2{\mathrm{O}}_2 + {\mathrm{e}}^{\hbox{-}}\to \cdotp \mathrm{O}\mathrm{H} + \mathrm{O}{\mathrm{H}}^{\hbox{-} } $$

8 $$ \cdotp \mathrm{O}\mathrm{H}+\mathrm{M}\mathrm{O}\to \mathrm{C}{\mathrm{O}}_2+{\mathrm{H}}_2\mathrm{O}+\mathrm{Others} $$

9 $$ \cdotp {{\mathrm{O}}_2}^{\hbox{-} }+\mathrm{M}\mathrm{O}\to \mathrm{C}{\mathrm{O}}_2+{\mathrm{H}}_2\mathrm{O}+\mathrm{Others} $$

10 $$ \cdotp \mathrm{O}\mathrm{O}\mathrm{H}+\mathrm{M}\mathrm{O}\to \mathrm{C}{\mathrm{O}}_2+{\mathrm{H}}_2\mathrm{O}+\mathrm{Others} $$

Therefore, the SrTiO_3_/TiO_2_ (rutile) composite nanofibers could be considered as an economical and continuable photocatalyst in future application.

## Conclusions

In summary, we have prepared the SrTiO_3_/TiO_2_ (rutile) composite nanofibers via a simple route of electrospinning and displayed its excellent ability to degrade methyl orange, which could be mainly ascribed to the remarkable heterojuction and the high crystallinity. What’s more, the novel 3D structure could increase the specific surface area efficiently, which is also an important reason for the photocatalysis. Thus excellent photocatalyst could afford a new sight for design of the future catalyst.
